# “A Re-Evaluation of M. prototuberculosis”: Continuing the Debate

**DOI:** 10.1371/journal.ppat.0020095

**Published:** 2006-09-29

**Authors:** Sylvain Brisse, Philip Supply, Roland Brosch, Veronique Vincent, M. Cristina Gutierrez

**Affiliations:** Scripps Research Institute, United States of America

In his Opinion [1], Noel Smith questions that the Mycobacterium tuberculosis complex (MTBC) is a successful clone that emerged from a much broader and ancient mycobacterial species, whose extant representatives are Mycobacterium canettii and other smooth tubercle bacilli [2]. He suggests that our conclusion is weak because he sees it as solely based on comparison of single nucleotide polymorphisms (SNPs) in six housekeeping genes and the 16 sRNA gene, and because some of these genes also showed evidence of horizontal transfer. Smith's criticism mostly comes down to two important assertions: first, MTBC members did not emerge from the smooth strains; second, the smooth strains and MTBC members should not be seen as a single species.

In response, we would stress that using comparison of SNP in housekeeping genes is now a widely accepted practice that has shown the clonal expansion of several other important bacterial pathogens from genetically broader progenitor species. Examples of such analyses are Yersinia pestis descended from Yersinia pseudotuberculosis [3], serotype Typhi from Salmonella enterica [4], *Shigella* clones from Escherichia coli [5], or Bordetella pertussis from Bordetella bronchiseptica [6]. For *M. tuberculosis,* Musser and colleagues [7] used SNP analysis of six housekeeping genes to detect the extremely reduced genetic polymorphisms that exist within the classical members of the MTBC. Most importantly, their results were later confirmed by whole genome comparisons [8–10]. It was therefore particularly exciting to find a much higher rate of sequence polymorphisms in the same six housekeeping genes that were used by Musser and colleagues when we studied smooth tubercle bacilli isolated in East Africa from patients with clinical tuberculosis, and these data represented the backbone of our published analysis [2]. However, we re-emphasize here that the greater genetic polymorphism observed among the smooth tubercle bacilli is not restricted to the six tested housekeeping genes, but also applies to other regions of the genomes, including conventionally used markers for differentiation of tubercle bacilli. These results, shown in supporting Figure S1 of our article [2], demonstrate that the smooth tubercle bacilli exhibited several outstanding features that underline their differences among each other and with the MTBC members. The number of IS*1081* copies constitutes one example. This insertion sequence has been so far described as being specific for tubercle bacilli [11]. Their number is highly conserved among all members of the MTBC, which carry five or six copies [11]. However, the smooth tubercle bacilli showed zero to three copies [2], indicating that this specific IS element was present in most strains of the smooth tubercle bacilli, but with a distinct copy number. In previous studies, the unusually low copy number of IS*1081* was also used to differentiate the smooth tubercle bacillus M. canettii from the classical MTBC members [12,13]. Other examples illustrating the wider diversity of smooth tubercle bacilli, mentioned in Protocol S2 and Figure S1, include the variable presence of another insertion sequence specific to smooth bacilli (IS*MycA1*), different MIRU–VNTR variant alleles also uniquely detected in smooth bacilli, and the absence of the direct repeat region in some smooth groups. In a different study, it was also shown that M. canettii harbored a completely different set of spacer sequences in the direct repeat region that was not present in the classical MTBC members [14]. Finally, Figure 1 shows our unpublished sequences of the 5′ end of the 16S rRNA gene in different lineages of the smooth tubercle bacilli. This region, which is 100% identical within the classical members of the MTBC, shows SNP variations that were only found within the smooth tubercle bacilli. This comparison also nicely shows that some lineages of smooth tubercle bacilli differ from others in an otherwise highly conserved region. Thus, altogether, our data clearly show that smooth tubercle bacilli harbor more diversity than MTBC members alone, although they share many characteristics in common (see below). More insight in this matter will certainly be provided by genome sequencing projects on M. canettii (http://www.sanger.ac.uk/sequencing/Mycobacterium/canetti) and other lineages of smooth tubercle bacilli.

**Figure 1:**
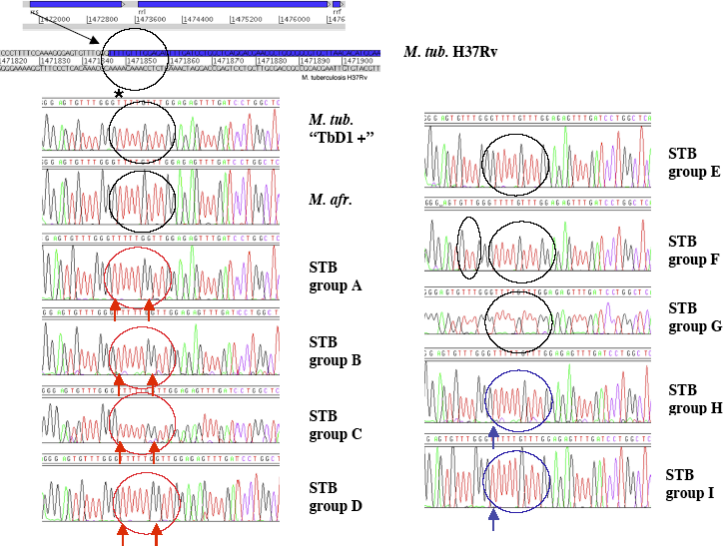
Polymophisms at the 5′ End of the 16S rRNA Gene in M. tuberculosis Complex Members and Smooth Tubercle Bacilli Four alleles are shown by colored circles. The allele of smooth groups H and I (AM283533) consists of a T insertion one nucleotide upstream of the 16S rRNA gene, shown by an arrow; the allele of smooth groups A, B, C, and D (AM283530) consists of the same T insertion and a G to T mutation at nucleotide 6 of the gene (corresponding to the mutation taken into account for the dendrogram shown in Figure 1 in our original article), both shown by an arrow; the allele of smooth group F (AM283532) consists of a T deletion (circled) further upstream of the gene. The allele of smooth groups E and G are identical at the 5′ locus to that of MTBC members (AM283534), but smooth group E (AM283531) has one T to C polymorphism at nucleotide 1017 of the 16S rRNA gene (not shown). The first nucleotide of the 16S rRNA gene is indicated by a star, above the M. tuberculosis TbD1+ sequence. M. afr., Mycobacterium africanum; M. tub, M. tuberculosis; STB, smooth tubercle bacilli.

In the case of the smooth tubercle bacilli strains, we demonstrated gene mosaicism, which we interpret as the result of homologous DNA recombination among smooth bacilli strains. Taking a different view, Smith speculates that the sequence diversity of the smooth strains, and their divergence from the MTBC members, mostly results from horizontal genetic transfer (HGT) into the smooth strains of DNA segments from donor strains of another species that remains to be discovered. Under this hypothesis, MTBC members would have been left untouched by HGT, whereas smooth strains would have been targeted with high frequency by HGT. Obviously, microbiologists are far from having discovered even a tiny fraction of existing biodiversity, and the existence of yet undiscovered mycobacterial species is almost certain. Noel Smith's hypothesis can therefore not be ruled out on the basis that the donor strains have not been discovered. Acquisition of some gene clusters through interspecies HGT has been recently well-described for mycobacteria [15–17], but such interspecies events can be readily recognized by specific genetic signatures. In our case, the levels of nucleotide identity of the recombinant segments in the smooth bacilli with the corresponding MTBC sequences is so high that the hypothetical donor of these recombinant segments would need to be much more closely related to the MTBC members than to any other mycobacterial species known so far (Table 1). Actually, our colleague's hypothesis would require the existence of a donor group that is as closely related (1% to 2% nucleotide divergence in housekeeping genes) to the MTBC members as are strains within single bacterial species [18]. With such a high degree of sequence identity, we think that it is more parsimonious to invoke homologous recombination within a single tubercle bacilli species, and do not see the need to invoke HGT from a hypothetical donor group of phylogenetically very closely related mycobacteria. In addition, our data on insertion sequences, direct repeat region spacers, and genomic deletions would not easily fit in with Smith's hypothesis.

**Table 1:**
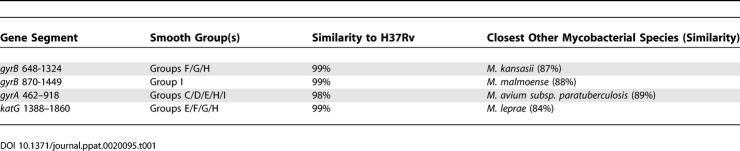
Level of Nucleotide Identities of Putative Recombinant Fragments of Smooth Tubercle Bacilli with the Corresponding Sequences of *M. tuberculosis* H37Rv, and with the Closest Mycobacterial Species (Excluding MTBC Members)

As reflected in his Opinion [1], Noel Smith does not accept the concept of M. prototuberculosis. Of course, the bacterial species concept and rules for species definition are subject to debate. To tackle these questions, recommendations have been issued by several international groups [19–21]. Although imperfect, one proposed species definition is pragmatic, operational, and universally applicable: “A species is a category that circumscribes a genomically coherent group of individual isolates/strains sharing a high degree of similarity in independent features (*note*: including phenotypic and clinical features)”. The use of nucleotide sequence divergence to define bacterial species is considered as one of the most promising approaches [19,20], and, when taken jointly with phenotypic and clinical parameters, should not necessarily be seen as “arbitrary and without merit.” In addition to common features in genotypical markers (see above), the smooth strains and MTBC were grouped into a compact phylogenetic clade remote from the closest mycobacterial species when we analyzed their phylogenetic relatedness with other mycobacterial species defined so far. The overall level of housekeeping gene nucleotide sequence similarity in this clade is as high as 98.46%, a level higher than observed in many current bacterial species. Moreover, smooth tubercle bacilli cause a disease whose clinical and radiological presentation is indistinguishable from tuberculosis caused by classical *M. tuberculosis,* but clearly distinct from diseases caused by other mycobacterial species. Because of this genetic and clinical coherence, we have proposed to give the name M. prototuberculosis to the phylogenetic entity constituted by the smooth strains (including M. canettii) and the MTBC members. Indeed, most smooth strain groups do not conform to all the formal criteria that define MTBC members and/or *M. canettii,* and a new name was therefore needed. Nevertheless, we want to make clear that we do not propose to abandon the usage of the current species names of the MTBC, such as M. tuberculosis or M. bovis. Nomenclature and evolutionary biology of bacteria have distinct premises and purposes, and distinct designations can coexist as long as the context in which they are used is clear. We thus propose to consider that the biological species M. prototuberculosis—which has no standing in nomenclature—comprises strains that conform to a set of nomenspecies with standing in nomenclature, such as M. tuberculosis. In the same way, for example, *Shigella* species or Y. pestis names are still retained in the nomenclature although is it known that they belong to a single genomic species together with E. coli or *Y. pseudotuberculosis,* respectively.

Noel Smith underlines the uncertainty inherent in the use of molecular clocks as well as methodological simplifications in our analysis. If one would accept his view that recombinant segments come from unrelated donor strains, then age estimates would be nonsense. However, under our hypothesis, the nucleotide diversity was generated within the M. prototuberculosis species, within which it has subsequently been shuffled among lineages. We agree that the age estimate may be overestimated because the available polymorphisms are not fixed in the population, and we certainly are aware of molecular clock uncertainties. We ourselves mentioned that the age scenario was speculative and put it forward with the main aim of challenging the widely accepted paradigm that tuberculosis is a recent disease of humans. In this sense, one should remember that the infectious cycle of the tubercle bacillus consists of a balance virtually unique among bacterial pathogens, between often lifelong asymptomatic parasitism and the development of tuberculous cavities in the lungs of the patient, which allows the efficient spread of high numbers of bacteria via aerosol to new hosts. From the perspective of this obligate pathogen, this balance is absolutely essential to maintain its so-successful life cycle, and it is very reasonable to think that this adaptation has evolved during a long-lasting co-evolution between the bacterium and its host. Therefore, our scenario offers a novel working frame to solve the apparent paradox between this exquisite adaptation and the previously hypothesized recent evolutionary origin of the pathogen [22] .

In summary, we thank Noel Smith for his critical comments, which have triggered many stimulating discussions and this response. We hope that we have clarified the different lines of evidence supporting our finding, as we are confident that the greater genetic diversity within this fascinating group of smooth tubercle bacilli is a key to a better understanding of the evolution of the major human pathogen M. tuberculosis.

## Supporting Information

### Accession Numbers

The EMBL (http://www.ebi.ac.uk/embl) accession numbers for the representative sequences discussed in Figure 1 are AM283530-AM283534.

## Abbreviations

HGT: horizontal genetic transfer
MTBC: Mycobacterium tuberculosis complex
SNP: single nucleotide polymorphism
